# Antidermatophytic Activity of Ethanolic Extract from* Croton tiglium*

**DOI:** 10.1155/2016/3237586

**Published:** 2016-07-03

**Authors:** Han Chien Lin, Yu-Liang Kuo, Wen-Ju Lee, Hui-Yi Yap, Shao-Hung Wang

**Affiliations:** ^1^Department of Wood-Based Materials and Design, College of Agriculture, National Chiayi University, Chiayi 60004, Taiwan; ^2^Department of Medical Imaging and Radiological Sciences, Chung Shan Medical University, Taichung 40201, Taiwan; ^3^Department of Medical Imaging, Chung Shan Medical University Hospital, Taichung 40201, Taiwan; ^4^Division of Cardiology, Department of Internal Medicine, Chi Mei Medical Center, Liouying, Tainan 73657, Taiwan; ^5^Department of Microbiology, Immunology and Biopharmaceuticals, National Chiayi University, Chiayi 60004, Taiwan

## Abstract

Dermatophytosis, which is caused mainly by genera of *Trichophyton*, *Epidermophyton*, and *Microsporum*, is a frequent dermatological problem in tropical and subtropical countries. Investigations were carried out in this study to evaluate the antidermatophytic activity of the stems, leaves, and seeds of *Croton tiglium*, one of the traditional medicine plants indigenous to Asia. Ethanolic extracts of the stems, leaves, and seeds of *C. tiglium* were prepared by cold soak or heat reflux methods. The antidermatophytic activities of the extracts were evaluated by disc diffusion and microdilution susceptibility assays against *Trichophyton mentagrophytes*, *T. rubrum*, and *Epidermophyton floccosum*. The active components in the extracts were analyzed and identified by GC-MS. All ethanolic extracts of *C. tiglium* showed some antifungal activities against the three dermatophytes. The ethanolic stem extract had the greatest inhibitory activities against *T. mentagrophytes* and *E. floccosum* with MICs at 0.16 mg/mL and had a lower activity against *T. rubrum* (MIC: 0.31 mg/mL). Oleic acid and hexadecanoic acid were found to be the major constituents in the stem extract that demonstrated strong antidermatophytic activities. The ethanolic extracts of stem or seed of *C. tiglium* exhibit strong antidermatophytic activities and, thus, could be considered for application on treating skin fungal infections after appropriate processing.

## 1. Introduction

Fungal infections of the skin and nails are common in humans and are caused mostly by dermatophytes. A microbiological surveillance demonstrated that molds actually account for a quarter of skin fungal pathogens in hospitalized fever patients in central Taiwan [1]. While the causative agents vary in species geographically,* T. mentagrophytes*,* T. rubrum*, and* E. floccosum* are the most common anthropophilic dermatophytes in Taiwan [2, 3]. Essential oils or organic extracts from plants have been reported with antidermatophytic activity, including* Abies holophylla*,* Angelica major*,* Alpinia speciosa*,* Artemisia sieberi*,* Baccharis trimera*,* Chamaecyparis obtusa*,* Cinnamomum zeylanicum*,* Cymbopogon citratus*,* Eucalyptus smithii*,* Eugenia uniflora*,* Foeniculum vulgare*,* Ocimum gratissimum, Mentha piperita*,* Moringa oleifera*,* Olea europaea*,* Prunus armeniaca*,* Prunus dulcis*, and* Thymus schimperi* [3–13].

*Croton tiglium* L. (Euphorbiaceae) is a medicinal plant that is indigenous to China, India, and Southeast Asia [14]. Seeds, leaves, bark, and root of* C. tiglium* are used in traditional medicine to ease various illnesses, including dropsy, obstinate constipation, intestinal obstruction, lead poisoning, and high blood pressure [15]. Moreover, extracts of* C. tiglium* have been reported possessing antimicrobial activities. The tested microorganisms include* Bacillus subtilis*,* Escherichia coli*,* Pseudomonas aeruginosa*,* Pasteurella multocida*,* Staphylococcus epidermidis*,* S. aureus*,* Aspergillus niger*,* Candida albicans*,* Microsporum canis*,* Mucor mucedo*, and* Trichophyton rubrum* [16–20]. Croton oil, prepared from the seeds of* C. tiglium*, was used to treat some skin diseases, including ringworm [21, 22]. To find whether* C. tiglium* has antidermatophytic activity, we extracted stems, leaves, and seeds of the plant separately and analyzed the possible active components after the extracts were further resolved by gas-chromatography (GC) coupled with mass spectrometry (MS).

## 2. Materials and Methods

### 2.1. Extraction of* Croton tiglium*

Stems, leaves, and seeds of* Croton tiglium* L. taken from the plants at Fanlu countryside, Chiayi county, Taiwan, were crushed and grinded. The powder was then extracted with ethanol either by cold soak or by heat reflux. In the cold soak method, 95% ethanol at a 10 : 1 weight ratio was used to soak the powder in dark at room temperature for 48 h. On the other hand of using heat reflux, the mixture of ethanol and powder was boiled and refluxed for 2 h in a flask equipped with a condenser. The filtrates (Advantec number 1) were concentrated and dried at 40°C in a rotary evaporator (Laborota 4000, Heidolph, Germany). After dissolving in a minimum amount of dimethyl sulfoxide, these extracts were used for the initial bioassay.

Additional dried ethanolic extracts were subjected to water and ethyl acetate partition (1 : 1) at room temperature. The ethyl acetate soluble fraction and the ethyl acetate insoluble but water soluble fraction were concentrated and dried at 40°C.

### 2.2. Fungal Strains and Culture Conditions

The dermatophytes used for experimental controls in this study were obtained from the Food Industry Research and Development Institute in Taiwan.* Trichophyton mentagrophytes* (BCRC 32066),* T. rubrum* (BCRC 32805), and* Epidermophyton floccosum* (BCRC 30531) were regularly cultured on Sabouraud dextrose agars (SDA; Difco) at 25°C. RPMI-1640 (Sigma) buffered to pH 7.0 with MOPS was used for the broth microdilution susceptibility assay, to determine the minimum inhibitory concentration (MIC). Amphotericin B (AMB; Sigma) was used as a positive control for antifungal activity. All the works were performed in biosafety level-2 (P2) facility. The protocol and the procedures of the study were approved by the National Chiayi University Biosafety Committee.

### 2.3. Antifungal Disc Diffusion Susceptibility Testing

Stock fungal conidia were prepared from 15-day-old colonies grown on potato dextrose agar (PDA; Difco) and suspended in sterile saline as a stock. SDA plates were streaked evenly with 0.5-McFarland inoculums. Samples to be tested were dissolved appropriately, and 10 *μ*L of the solution was applied to sterile paper discs (ADVANTEC), 8 mm in diameter, and air dried. The discs were then applied to the surfaces of inoculated plates. Plates were incubated at 25°C for 48 h, and the diameters of the inhibition zone were measured.

### 2.4. Susceptibility Assay by Broth Microdilution

MICs of the samples were determined alternatively by the broth microdilution method using serially diluted extract samples. In brief, each microdilution well that contained 100 *μ*L of the MOPS-buffered RPMI-diluted extract received an equal volume of 50-fold diluted conidia stock. The well-containing plates were incubated at 25°C for 48 h. After incubation, the plates were examined visually, and the MIC for the assayed sample was the lowest extract concentration showing no apparent growth of the target microorganism.

### 2.5. Identification of Components in the Ethyl Acetate Soluble Fractions

To analyze the components in the ethyl acetate soluble fractions of the* C. tiglium*, GC-MS (Agilent model 6890 GC with model 5973 MSD) was applied. To DB-5ms column (30 m × 0.25 mm i.d. × 0.25 *μ*m film thickness), 1 *μ*L sample was injected. The oven temperature was set initially at 40°C for 1 min, followed by an increment of 4°C/min up to 180°C, then by an increase of 15°C/min up to 260°C, and then by keeping at 260°C for 10 min; the injector was set at 270°C with helium as the carrier gas (1 mL/min). The split valve was opened with a split ratio of 1 : 50 and the ionization energy was 70 eV. Spectra were scanned from 45 to 425 m/z. Volatile compounds were identified by comparing the mass spectra with the data system libraries (Wiley/NBS and NIST) and by Kovats indices (KI) estimated in accordance with modified Kovats method [23]. The relative percentages of individual compounds were calculated from the corresponding peak areas.

### 2.6. Statistical Analysis

Experimental measurements were carried out in triplicate. All values are expressed as means ± standard deviation. Statistical analysis was performed using one-way analysis of variance followed by Duncan's multiple comparison test using Statistical Product and Service Solutions (SPSS). Differences were considered statistically significant when *p* < 0.05.

## 3. Results

### 3.1. Antidermatophytic Activity of Ethanolic Extract from* Croton tiglium*

Three most common dermatophytes in Taiwan, that is,* T. mentagrophytes*,* T. rubrum*, and* E. floccosum*, were used for testing the antifungal activity of* C. tiglium* extracts in this study (Tables 1 [Table tab2]–3). By using disc diffusion method, we demonstrated that both heat reflux and cold soak fractions of the ethanolic stem extract of* C. tiglium* had inhibitory activities against* T. mentagrophytes* (Table 1) and* E. floccosum* (Table 3) in a dose-dependent manner.* T. rubrum* is less sensitive to all of the extracts. However, when the heat reflux extract was used at 10 *μ*g (Table 2), a clear zone can be seen. The heat reflux or the cold soak fractions of* C. tiglium* seeds showed a weak but noteworthy inhibition against* T. mentagrophytes* (Table 1) and* E. floccosum* (Table 3). It is conclusively seen that using the heat reflux method to extract stems of* C. tiglium* yielded the highest inhibition activity to the three dermatophytes among all preparations (Tables 1 and 3).

To determine the MICs of those extracts against the three dermatophytes, broth microdilution susceptibility was performed. The smallest values of MIC of the prepared* C. tiglium* extracts against* T. mentagrophytes*,* T. rubrum*, and* E. floccosum* were 0.16 mg/mL, 0.31 mg/mL, and 0.16 mg/mL, respectively. They were all obtained from the heat reflux-derived extracts of stem (Table 4), an observation that was consistent with the inhibitory results using disc diffusion method (Tables 1 and 3).

### 3.2. Antidermatophytic Activity of the Ethyl Acetate Fractions from* Croton tiglium* Ethanolic Extracts

To analyze the antidermatophytic ingredients in the extracts of* C. tiglium*, the preparations were partitioned with water and ethyl acetate. The ethyl acetate soluble parts, but not the water soluble fraction (i.e., ethyl acetate insoluble fractions), readily possessed antidermatophytic activity (Table 5). Moreover, the values of MIC measured for the ethyl acetate soluble fractions were all smaller than the original nonpartitioned ones (Tables 4 and 5). Among them, those ethyl acetate extracts from the heat reflux of the* C. tiglium* stem showed the strongest antidermatophytic activity (Table 5) with the values of MIC against* T. mentagrophytes*,* T. rubrum*, and* E. floccosum* at 0.08 mg/mL, 0.16 mg/mL, and 0.04 mg/mL, respectively. Second to the stem preparations, the counterparts prepared from the heat reflux seed showed MIC strengths at 0.16 mg/mL, 0.31 mg/mL, and 0.16 mg/mL, respectively (Table 5). Those from the leaves yielded the least inhibitory activities.

### 3.3. Component Identification of the Ethyl Acetate-Partitioned, Heat Reflux Ethanolic Extracts from* C. tiglium*

To examine what compounds within the* C. tiglium* extracts have the antidermatophytic activities, the final ethyl acetate preparations from the heat reflux ethanolic extracts of* C. tiglium* stem and seed were separately subjected to GC-MS analysis. In the GC profiles, any peak with a volume higher than 1% of the total peak areas was examined and their components were identified by matching to the built-in data.  Table 6 shows ten major components in the stem extract while showing eight components in the seed preparation. Among the ten major components in the stem extract, one (hexadecanoic acid, ethyl ester) was also detected abundantly in the seed extract. The top four abundant compounds in the stem extract were oleic acid, ethyl ester (20.87%), hexadecanoic acid (palmitic acid, 20.77%), hexadecanoic acid, ethyl ester (14.11%), and oleic acid (14.04%).

### 3.4. Antidermatophytic Activity of Oleic Acid

Since the major chemicals identified in the ethyl acetate extracts of* C. tiglium* stem and seed were long-chain organic acids or their esters (Table 6), we tested the top four major components found in the ethyl acetate-stem extract for their possible antifungal activities by the microdilution method, after balancing the pH values. The results in Table 7 show that oleic acid (MIC: 0.08–0.31 mg/mL) is the most active one, followed by hexadecanoic acid (MIC: 0.16–0.31 mg/mL), against the three common dermatophytes. In contrast, their ethyl esters showed less active (oleic acid, ethyl ester with MIC at 0.63 to >5 mg/mL) or negligible activities (hexadecanoic acid, ethyl ester with MIC at >5 mg/mL). As aforementioned,* T. rubrum* displayed less sensitivity to those compounds than the other two fungi.

## 4. Discussion

*C. tiglium*, a plant considered indigenous to Asia, has been used long time for medical purposes. In the present study, we revealed that the ethanolic extract of* C. tiglium* did possess strong antifungal activities against dermatophytes, including* T. mentagrophytes*,* T. rubrum*, and* E. floccosum*. GC-MS analysis revealed that the major components in the stem or seed extracts of* C. tiglium* were long-chain organic acids and their ester derivatives. Moreover, we found that oleic acid and hexadecanoic acid of the extracts, but not their ester forms, exhibited strong antidermatophytic activity.

In a screening of medicinal plants for the antifungal activity, Jamil et al. found that crude extracts from the leaves and seeds of* C. tiglium* were active against Trichocomaceae (*Aspergillus niger* and* A. tamarii*) and Mucoraceae (*Mucor mucedo* and* Rhizopus solani*) [24]. In a separate experiment, Shahid et al. discovered that a 50 kDa protein had good antifungal activity [18]. However, Iraqui and Yadav [19] have discovered the ethanolic or methanolic extracts from seeds of* C. tiglium* exhibiting better fungicidal activity against* T. rubrum* than the water extracts. Here we consistently found that the portion of* C. tiglium* ethanol extract followed by partition into ethyl ester did contain the anti-*T. rubrum* activities. However, we did not aim at the large molecules solubilized in water. The possible antifungal protein in* C. tiglium* remains to be confirmed. It is conceivable that ingredients of* C. tiglium* against dermatophytes may consist of multiple components.

Extracts of leaves from* Helichrysum pedunculatum* have been previously proven to be active against Gram-positive bacteria and within the extracts, oleic and linoleic acids are the active constituents. Furthermore, they have synergistic bactericidal activity against* Staphylococcus aureus* and* Micrococcus kristinae* [25]. Linolenic, linoleic, and oleic acids have been demonstrated to exhibit strong activities against some important plant fungal pathogens [26]. In our case of* C. tiglium*, high contents of oleic acid and hexadecanoic acid (palmitic acid) seem to play a major role in inhibiting three human fungal pathogens. Intriguingly, polyunsaturated fatty acids seem to be more active against dermatophytes than the saturated fatty acid [27] as the effective linoleic (C 18:2), linolenic (C 18:3), and oleic (C 18:1) acids are all unsaturated.

## 5. Conclusion

The ethanolic extracts of stem or seed extracts of* C. tiglium* exhibited strong antidermatophytic activities. A topical application of the ethanolic extracts of* C. tiglium* on treating skin fungal infection and formulation of the extracts into shampoo or soap may be practical and scientifically sounding.

## Figures and Tables

**Table 1:** Antifungal activity of *Croton tiglium* ethanolic extracts against *Trichophyton mentagrophytes* by the disc diffusion method^*∗*^.

Specimen		Stem		Leave		Seed	
		Heat reflux	Cold soak	Heat reflux	Cold soak	Heat reflux	Cold soak
Extract	10 *μ*g	11.1 ± 0.4^za^	8.7 ± 0.3^xa^	8.0 ± 0.0^wa^	8.0 ± 0.0^wa^	9.5 ± 0.4^ya^	9.1 ± 0.3^xya^
	50 *μ*g	14.0 ± 0.4^yb^	11.7 ± 0.2^xb^	9.6 ± 0.3^wb^	8.7 ± 0.5^wa^	13.0 ± 0.6^yb^	11.7 ± 0.6^xb^
	250 *μ*g	17.0 ± 0.2^zc^	14.2 ± 0.6^yc^	12.5 ± 0.2^xc^	11.1 ± 0.2^wb^	16.1 ± 0.8^zc^	14.4 ± 0.7^yc^
	500 *μ*g	18.1 ± 0.4^yd^	15.2 ± 0.4^xd^	13.4 ± 0.1^wd^	12.4 ± 0.8^wc^	17.3 ± 0.7^yc^	15.4 ± 0.8^xc^

AMB (10 *μ*g)		21.3 ± 0.2^e^					

^*∗*^The fungal spores were plated on SDA plates with the extracts or amphotericin B (AMB) absorbed on 8 mm filter paper discs. Duncan's multiple range test (*p* < 0.05) was used to statistically evaluate the difference in the diameters (mm) of inhibition zones. The letters “a, b, c, d, and e” indicated significant differences between groups treated with different amounts of the extracts. The letters “w, x, y, and z” indicate statistical differences among the extraction groups.

**Table 2 tab2:** Antifungal activity of *Croton tiglium* ethanolic extracts against *Trichophyton rubrum* by the disc diffusion method^*∗*^.

Specimen		Stem		Leave		Seed	
		Heat reflux	Cold soak	Heat reflux	Cold soak	Heat reflux	Cold soak
Extract	10 *μ*g	9.8 ± 0.5^xa^	8.0 ± 0.0^wa^	8.0 ± 0.0^wa^	8.0 ± 0.0^wa^	8.0 ± 0.0^wa^	8.0 ± 0.0^wa^
	50 *μ*g	13.9 ± 0.1^yb^	10.1 ± 0.4^xb^	8.9 ± 0.2^wb^	9.9 ± 0.2^wxb^	10.0 ± 0.2^xb^	10.0 ± 1.0^xb^
	250 *μ*g	16.8 ± 0.1^yc^	12.5 ± 0.3^xc^	10.5 ± 0.2^wc^	12.3 ± 0.1^xc^	12.9 ± 0.4^xc^	12.7 ± 0.9^xc^
	500 *μ*g	17.7 ± 0.5^yd^	13.5 ± 0.21^xd^	11.8 ± 0.1^wd^	13.0 ± 0.2^xd^	13.8 ± 0.6^xd^	13.4 ± 0.5^xc^

AMB (10 *μ*g)		20.2 ± 0.2^e^					

^*∗*^The notes are the same as those in Table 1.

**Table 3:** Antifungal activity of *Croton tiglium* ethanolic extracts against *Epidermophyton floccosum* by the disc diffusion method^*∗*^.

Specimen		Stem		Leave		Seed	
		Heat reflux	Cold soak	Heat reflux	Cold soak	Heat reflux	Cold soak
Extract	10 *μ*g	12.04 ± 0.65^wa^	10.08 ± 0.41^xa^	8.50 ± 0.35^vwa^	8.00 ± 0.00^va^	8.88 ± 0.27^wa^	8.00 ± 0.00^va^
	50 *μ*g	15.08 ± 0.72^xb^	13.88 ± 0.53^wxb^	10.29 ± 1.10^vb^	9.42 ± 0.24^vb^	12.79 ± 0.60^wb^	10.79 ± 0.06^vb^
	250 *μ*g	17.50 ± 0.20^zc^	16.08 ± 0.33^yc^	12.42 ± 0.36^vc^	13.50 ± 0.35^wc^	15.17 ± 0.24^xc^	13.92 ± 0.31^wc^
	500 *μ*g	18.29 ± 0.29^xc^	17.08 ± 0.46^wc^	15.04 ± 0.91^vd^	14.25 ± 0.54^vc^	16.38 ± 0.18^wd^	14.92 ± 0.51^vd^

AMB (10 *μ*g)		21.4 ± 0.1^e^					

^*∗*^The notes are the same as those in Table 1.

**Table 4:** Minimal inhibitory concentrations (mg/mL) of *Croton tiglium* ethanolic extracts against dermatophytes.

Specimen		*T. mentagrophytes*	*T. rubrum*	*E. floccosum*
Stem	Heat reflux	0.16	0.31	0.16
	Cold soak	0.63	1.25	0.31

Leave	Heat reflux	1.25	2.50	0.63
	Cold soak	2.50	1.25	1.25

Seed	Heat reflux	0.31	1.25	0.63
	Cold soak	0.63	1.25	1.25

**Table 5:** Minimal inhibitory concentrations (mg/mL) against dermatophytes of the ethyl acetate fraction of the *Croton tiglium* ethanolic extracts^*∗*^.

Specimen		*T. mentagrophytes*	*T. rubrum*	*E. floccosum*
Stem	Heat reflux	0.08	0.16	0.04
	Cold soak	0.31	0.63	0.16

Leave	Heat reflux	0.63	1.25	0.31
	Cold soak	1.25	0.63	0.63

Seed	Heat reflux	0.16	0.31	0.16
	Cold soak	0.31	0.63	0.31

^*∗*^All ethyl acetate insoluble (water soluble) fractions gave MIC values greater than 5 mg/mL.

**Table 6:** GC-MS identification of ethyl acetate soluble components of the heat reflux ethanolic extracts from *Croton tiglium*.

Compound identification		Stem	Seed
Esters	Bis (2-ethylhexyl) phthalate	5.23^*∗*^	—
	Decanoic acid, ethyl ester	—	6.44
	Dodecanoic acid, ethyl ester	—	5.74
	Hexadecanoic acid, ethyl ester	14.11	17.78
	Tetradecanoic acid, ethyl ester	—	4.39
	15-Methyl-11-hexadecenoic acid, methyl ester	1.87	—
	Heptadecanoic acid, ethyl ester	—	0.95
	Linoleic acid, ethyl ester	—	35.90
	Octadecanoic acid, ethyl ester	5.56	24.47
	Oleic acid, ethyl ester	20.87	—

Alcohols	Stigmasterol	—	4.33

Acids	Hexadecanoic acid	20.77	—
	Oleic acid	14.04	—

Alkanes	Dodecamethylcyclohexasiloxane	2.68	—
	Eicosane	7.90	—
	Tetradecamethyl-cycloheptasiloxane	1.49	—

^*∗*^Numbers indicate the percentages of each GC-MS peak. Those peaks with percentages less than 1% were omitted.

**Table 7:** Minimal inhibitory concentrations (mg/mL) against dermatophytes of the major components of the *Croton tiglium* stem heat reflux extracts.

	*T. mentagrophytes*	*T. rubrum*	*E. floccosum*
Oleic acid	0.08	0.31	0.08
Hexadecanoic acid	0.31	0.31	0.16
Oleate, ethyl ester	2.5	>5	0.63
Hexadecanoic acid, ethyl ester	>5	>5	>5
